# Associations between gestational weight gain and adverse neonatal outcomes: a comparison between the US and the Chinese guidelines in Chinese women with twin pregnancies

**DOI:** 10.1186/s12889-023-15008-z

**Published:** 2023-01-19

**Authors:** Feng Liang, Yun Lin, Ling Li, Chuanzi Yang, Xiaojun Li, Kuanrong Li

**Affiliations:** Clinical Data Center, Guangzhou Women and Children’s Medical Center, Guangzhou Medical University, No. 9 Jinsui Road, Zhujiang Newtown, Tianhe District, Guangzhou, 510623 China

**Keywords:** Gestational weight gain, Twin pregnancy, Neonatal outcome, IOM guidelines

## Abstract

**Background:**

Appropriate gestational weight gain (GWG) is essential for maternal and fetal health. For twin pregnancies among Caucasian women, the Institute of Medicine (IOM) guidelines can be used to monitor and guide GWG. We aimed to externally validate and compare the IOM guidelines and the recently released guidelines for Chinese women with twin pregnancies regarding the applicability of their recommendations on total GWG (TGWG).

**Method:**

A retrospective cohort study of 1534 women who were aged 18–45 years and gave birth to twins at ≥ 26 gestational weeks between October 2016 and June 2020 was conducted in Guangzhou, China. Women's TGWG was categorized into inadequate, optimal, and excess per the IOM and the Chinese guidelines. Multivariable generalized estimating equations logistic regression was used to estimate the risk associations between TGWG categories and adverse neonatal outcomes. Cohen’s Kappa coefficient was calculated to evaluate the agreement between the IOM and the Chinese guidelines.

**Results:**

Defined by either the IOM or the Chinese guidelines, women with inadequate TGWG, compared with those with optimal TGWG, demonstrated higher risks of small-for-gestational-age birth and neonatal jaundice, while women with excess TGWG had a higher risk of delivering large-for-gestational-age infants. The agreement between the two guidelines was relatively high (Kappa coefficient = 0.721). Compared with those in the optimal TGWG group by both sets of the guidelines, women classified into the optimal group by the Chinese guidelines but into the inadequate group by the IOM guidelines (*n* = 214) demonstrated a statistically non-significant increase in the risk of all the adverse neonatal outcomes combined.

**Conclusions:**

The IOM and the Chinese guidelines are both applicable to Chinese women with twin pregnancies.

**Supplementary Information:**

The online version contains supplementary material available at 10.1186/s12889-023-15008-z.

## Background

Women pregnant with twins are at higher risks of adverse neonatal outcomes than women with singleton pregnancies [1–3]. Maternal gestational weight gain (GWG) is a valuable albeit simple indicator for newborn morbidities. However, recognition of abnormal GWG is a challenging task that requires reliable assessment tools.

In 2009, the Institute of Medicine (IOM) issued guidelines on total GWG (TGWG) for twin pregnancies in Caucasian women [4]. Taking into account women's prepregnancy body mass index (PBMI) status by the WHO criteria, the IOM recommends TGWG of 17.0–25.0 kg for normal-weight women (PBMI 18.5–24.9 kg/m^2^), 14.0–23.0 kg for overweight women (PBMI 25.0–29.9 kg/m^2^), and 11.0–19.0 kg for obese women (PBMI ≥ 30.0 kg/m^2^) [4]. Studies in Western populations have found that inappropriate *vs.* optimal TGWG, as defined by the IOM guidelines, is associated with increased risks of unhealthy birthweight and neonatal intensive care unit (NICU) admission [5–8]. Despite the ethnic disparity, several studies have also appraised the appropriateness of the IOM guidelines in Asian populations [9–11], producing largely similar results.

National guidelines on TGWG for twin pregnancies have not been formulated in many Asian countries including China, although some regional guidelines based on less representative data have been made available [11, 12]. Based on data from a regional but relatively large population, the recent Chinese guidelines recommend TGWG of 18.0–26.0 kg for underweight women (PBMI < 18.5 kg/m^2^), 15.0–25.0 kg for normal-weight women (PBMI 18.5–23.9 kg/m^2^, according to the Chinese criterion), 12.0–21.0 kg for overweight women (PBMI 24.0–27.9 kg/m^2^), and 9.0–20.0 kg for obese women (PBMI ≥ 28.0 kg/m^2^) [11]. It is apparent that the Chinese guidelines tend to recommend lower TGWG than do the IOM guidelines, and this is particularly the case for women who would be categorized into different PBMI groups by the inconsistent PBMI cut-offs. It is also notable that the Chinese guidelines include a recommendation for underweight women.

At this stage, it is important to validate and compare the IOM and the Chinese guidelines, which will facilitate decision-making on which guidelines to choose. Here, we reported a retrospective cohort study that was conducted particularly for this purpose.

## Methods

### Study design and data source

**Fig. 1:**
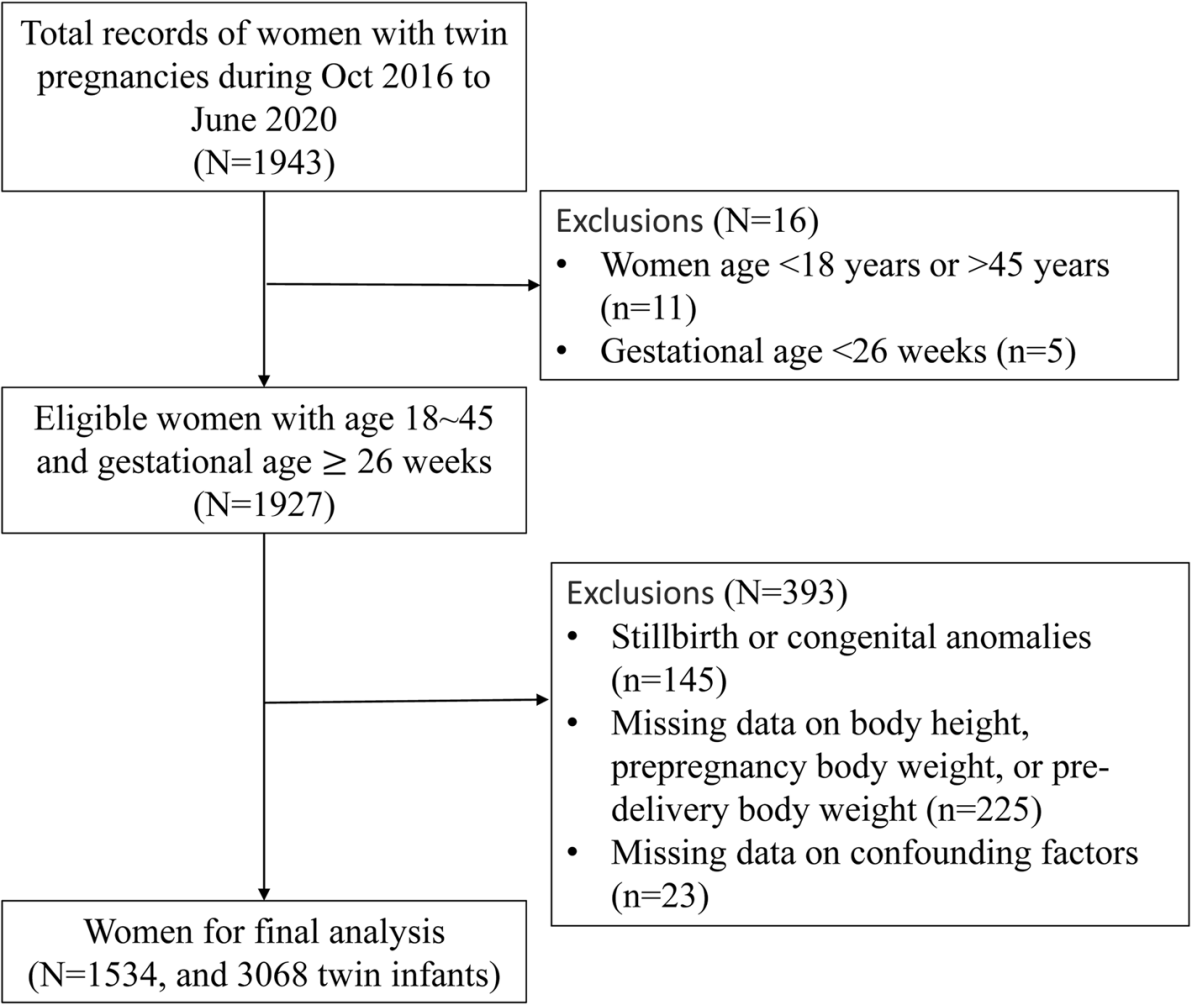
Flowchart of study participant selection

A retrospective cohort study was designed to include all women who gave birth to twins between October 2016 and June 2020 in Guangzhou Women and Children’s Medical Center, a tertiary medical facility in South China. Information on demographics, reproductive history, and maternal and neonatal factors relative to the current pregnancies was retrieved from clinical records. Eligible women were those who were aged 18 − 45 years and gave birth to twins at ≥ 26 gestational weeks during the five-year period (n = 1927). Women who delivered stillbirths or births with congenital anomalies (*n* = 145) were excluded. Women with missing data on the following key variables were excluded: body height, prepregnancy body weight, pre-delivery body weight, and other confounding factors including maternal age, gravidity, parity, historical cesarean section, education level, use of assisted reproductive technology (ART), twin type, pre-existing diabetes/hypertension, gestational diabetes mellitus (GDM), gestational hypertension, and family history of diabetes/hypertension. These exclusion criteria eventually led to a final cohort of 1534 women for analysis (Fig. 1).

### Ascertainment of total gestational weight gain and adverse neonatal outcomes

Maternal prepregnancy body weight was self-reported at the first prenatal care visit. If possible, missing values on prepregnancy body weight were substituted with body weight measured in the first 12 weeks of gestation. Pre-delivery body weight was recorded at the admission for hospital delivery. TGWG was calculated as the difference between pre-delivery and prepregnancy body weights. In accordance with the IOM guidelines, we categorized women into inadequate, optimal, and excess TGWG groups. The same groups were formed again using the Chinese guidelines.

This study considered the following adverse neonatal outcomes: small for gestational age (SGA), large for gestational age (LGA), respiratory distress syndrome, neonatal jaundice, and NICU admission. Nationwide surveillance data were used to identify SGA and LGA, which referred to birth weight below the 10th and above the 90th percentile references, respectively [13]. Respiratory distress syndrome was defined as the presence of one of the following signs: tachypnea (respiratory rate > 60 breaths/minute), grunting, nasal flaring, or intercostal retraction [14]. Neonatal Jaundice was diagnosed following the criteria of National Institute for Health and Care Excellence [15].

### Statistical analysis

Maternal and neonatal characteristics were described using median and inter-quartile range (IQR) for continuous variables and frequency for categorical variables. Multivariable logistic regressions based on general estimated equations (GEE) were used to estimate the adjusted odds ratios (aORs) of adverse neonatal outcomes for inadequate and excess *vs.* optimal TGWG after controlling for maternal age, gestational age, PBMI, gravidity, parity, historical cesarean section, educational level, use of ART, twin type, pre-existing diabetes/hypertension, GDM, gestational hypertension, and family history of diabetes/hypertension. Cohen’s Kappa coefficient was calculated to evaluate the agreement between the IOM and the Chinse guidelines. Two-sided *P*-value < 0.05 was considered statistically significant. All the statistical analyses were performed using R version 4.0.2 (R Foundation for Statistical Computing, Vienna, Austria).

## Results

The characteristics of the study population are summarized in Table 1. The median maternal age was 31.0 years (IQR 29.0–34.0). A majority of women (85.3%) had a college degree. Nulliparous and primigravid women accounted for 61.0% and 44.3% of the study population, respectively. The prevalence of prepregnancy underweight, normal weight, overweight, and obesity, as defined by Chinese BMI criteria, was 16.0%, 67.5%, 12.9%, and 3.6%, respectively. More than half (54.2%) of women conceived via ART. About 37.7% of parous women had historical cesarean section, 15.4% had family history of diabetes mellitus/hypertension, 1.9% had pre-existing diabetes mellitus/hypertension, 24.6% had GDM and 16.5% had gestational hypertension. A majority of women (93.9%) chose cesarean section. The percentages of dichorionic, monochorionic-diamniotic, monoamniotic twin pregnancies were 74.2%, 25.4% and 0.5%, respectively. The median gestational age at delivery was 37.0 weeks (IQR: 35.0–37.0). Of the 3068 twin infants, 1609 (52.4%) were male. We further stratified the study population into inadequate, optimal, and excess TGWG groups using the IOM and the Chinese guidelines and reported their characteristics in Table S1.

**Table 1:** Baseline characteristics of 1534 women with twin pregnancies

**Characteristics**	
Maternal age, year, median [IQR]	31.0 [29.0, 34.0]
Gestational age, weeks, median [IQR]	37.0 [35.0, 37.0]
Education level, n (%)	
Primary school or middle school	18 (1.2)
High school	207 (13.5)
College	1309 (85.3)
Nulliparity, n (%)	
No	599 (39.0)
Yes	935 (61.0)
Primigravida, n (%)	
No	854 (55.7)
Yes	680 (44.3)
Categorical PBMI by the Chinese criteria, n (%)	
Underweight (< 18.5 kg/m^2^)	245 (16.0)
Normal (18.5–23.9 kg/m^2^)	1036 (67.5)
Overweight (24.0–27.9 kg/m^2^)	198 (12.9)
Obese (≥ 28.0 kg/m^2^)	55 (3.6)
Categorical PBMI by the WHO criteria, n (%)	
Underweight (< 18.5 kg/m^2^)	245 (16.0)
Normal (18.5–24.9 kg/m^2^)	1108 (72.2)
Overweight (25.0–29.9 kg/m^2^)	158 (10.3)
Obese (≥ 30.0 kg/m^2^)	23 (1.5)
Use of ART, n (%)	
No	703 (45.8)
Yes	831 (54.2)
Historical cesarean section, n (%)^a^	
No	373(62.3)
Yes	226(37.7)
Family history of diabetes mellitus/hypertension, n (%)	
No	1298 (84.6)
Yes	236 (15.4)
Pre-existing diabetes mellitus/hypertension, n (%)	
No	1505 (98.1)
Yes	29 (1.9)
GDM, n (%)	
No	1157 (75.4)
Yes	377 (24.6)
Gestational hypertension, n (%)	
No	1281 (83.5)
Yes	253 (16.5)
Delivery mode, n (%)	
Cesarean section	1440 (93.9)
Vaginal delivery	94 (6.1)
Twin type, n (%)	
Dichorionic	1138 (74.2)
Monochorionic-diamniotic	389 (25.4)
Monoamniotic	7 (0.5)
Offspring sex, n (%)	
Male	1609 (52.4)
Female	1459 (47.6)

*Abbreviations: ART* assisted reproductive technology, *GDM* gestational diabetes mellitus, *GWG* gestational weight gain, *IQR* inter-quartile range, *PBMI*, prepregnancy body mass index, *WHO* World Health Organization

^a^The percentage of historical cesarean section was calculated in 599 parous women

Risk associations between TGWG categories and adverse neonatal outcomes are presented in Table 2. Defined by either IOM or the Chinese guidelines, inadequate TGWG, as compared with the optimal TGWG, was associated with statistically significantly increased risks of SGA (aOR_CHN_ = 1.66, 95% CI: 1.27–2.18; aOR_IOM_ = 1.67, 95% CI:1.23–2.27), neonatal jaundice (aOR_CHN_ = 1.34, 95%CI: 1.10–1.64; aOR_IOM_ = 1.31, 95% CI: 1.05–1.63), and occurrence of any adverse neonatal outcome (aOR_CHN_ = 1.37, 95% CI: 1.13–1.64; aOR_IOM_ = 1.28, 95% CI: 1.06–1.56). Excess TGWG defined by either the IOM or the Chinese guidelines was associated with an increased risk of LGA (aOR_CHN_ = 4.58, 95% CI: 2.85–7.38; aOR_IOM_ = 3.48, 95% CI: 1.97–6.13). As shown in Table S2, exclusion of underweight women (*n* = 245) for the Chinese guidelines did not change the statistical significance of the associations of inadequate TGWG with SGA, neonatal jaundice, and occurrence of any adverse neonatal outcome and the association of excess TGWG with LGA.

**Table 2:** Adjusted risk associations of neonatal outcomes for women with inadequate or excess TGWG *vs.* women with optimal TGWG, as defined by the Chinese or the IOM guidelines

**Outcome**	**Optimal TGWG**^b^	**Inadequate TGWG**		**Excess TGWG**	
	**n/N (%)**	**n/N (%)**	**aOR (95% CI)**^c^	**n/N (%)**	**aOR (95% CI)**^c^
**The Chinese guidelines **					
SGA	153/1714 (8.9)	166/1144 (14.5)	1.66 (1.27, 2.18)	9/210 (4.3)	0.43 (0.21, 0.89)
LGA	78/1714 (4.6)	25/1144 (2.2)	0.57 (0.35, 0.93)	36/210 (17.1)	4.58 (2.85, 7.38)
Respiratory distress	45/1714 (2.6)	104/1144 (9.1)	1.40 (0.81, 2.41)	9/210 (4.3)	1.74 (0.55, 5.52)
Neonatal jaundice	463/1714 (27.0)	433/1144 (37.8)	1.34 (1.10, 1.64)	51/210 (24.3)	0.81 (0.54, 1.24)
NICU admission	119/1714 (6.9)	144/1144 (12.6)	0.69 (0.45, 1.06)	6/210 (2.9)	0.27 (0.09, 0.86)
Any adverse outcome^a^	637/1714 (37.2)	584/1144 (51.0)	1.37 (1.13, 1.64)	91/210 (43.3)	1.30 (0.91, 1.85)
**The IOM guidelines**					
SGA	91/1116 (8.2)	165/1302 (12.7)	1.67 (1.23, 2.27)	5/160 (3.1)	0.35 (0.14, 0.87)
LGA	64/1116 (5.7)	35/1302 (2.7)	0.47 (0.30, 0.75)	25/160 (15.6)	3.48 (1.97, 6.13)
Respiratory distress	25/1116 (2.2)	106/1302 (8.1)	1.52 (0.74, 3.14)	8/160 (5.0)	2.72 (0.80, 9.25)
Neonatal jaundice	287/1116 (25.7)	459/1302 (35.3)	1.31 (1.05, 1.63)	39/160 (24.4)	0.87 (0.55, 1.40)
NICU admission	70/1116 (6.3)	167/1302 (12.8)	0.87 (0.55, 1.36)	4/160 (2.5)	0.21 (0.02, 1.89)
Any adverse outcome ^a^	399/1116 (35.8)	627/1302 (48.2)	1.28 (1.06, 1.56)	68/160 (42.5)	1.26 (0.84, 1.89)

*Abbreviations: aOR* adjusted odds ratio, *ART* assisted reproductive technology, *CI* confidence interval, *GDM* gestational diabetes mellitus, *IOM* Institute of Medicine, *LGA* large for gestational age, *NICU* neonatal intensive care unit, *PBMI* prepregnancy body mass index, *SGA* small for gestational age, *TGWG* total gestational weight gain

^a^Any adverse outcome was defined as the presence of one or more of the following neonatal outcomes: SGA, LGA, respiratory distress syndrome, neonatal jaundice, and NICU admission

^b^Optimal TGWG was used as the reference

^c^All the models adjusted for the priori defined confounders, including maternal age, gestational age, maternal PBMI, parity, gravidity, education level, twin type, use of ART, historical cesarean section, family history of diabetes mellitus/hypertension, pre-existing diabetes mellitus/hypertension, GDM and gestational hypertension

Further comparison between the IOM and the Chinese guidelines was made by cross-classifying the TGWG using the two sets of the guidelines after excluding underweight women. Among the remaining 1289 women, a total of 1064 (82.5%) women were classified into the same categories by both sets of the guidelines(Kappa coefficient = 0.721, 95% CI: 0.687–0.755). Two hundred and fourteen (16.6%) women were classified into the optimal group by the Chinese guidelines but into the inadequate group by the IOM guidelines (Table 3). As shown in Table 4, compared with women in the optimal TGWG group by both sets of the guidelines, those in the optimal group by the Chinese guidelines but in the inadequate group by the IOM guidelines demonstrated a statistically significantly decreased LGA risk (aOR = 0.51, 95% CI: 0.26–0.99) but no statistically significant difference in the risk of any adverse neonatal outcome (aOR = 1.06, 95% CI: 0.81–1.40).

**Table 3:** The cross-classification of TGWG by the IOM and the Chinese guidelines

		IOM guidelines			
		Inadequate	Optimal	Excess	Total
Chinese guidelines	Inadequate	437 (33.9)	0	0	437 (33.9)
	Optimal	214 (16.6)	547 (42.4)	0	761 (59.0)
	Excess	0	11 (0.9)	80 (6.2)	91 (7.1)
	Total	651 (50.5)	558 (43.3)	80 (6.2)	1289 (100)

Data are n (%). Cohen’s Kappa coefficient = 0.721 (95% CI: 0.687–0.755)

*Abbreviations: CI* confidence interval, *IOM* Institute of Medicine, *TGWG* total gestational weight gain

**Table 4:** Adjusted risk associations of neonatal outcomes for women with adequate TGWG by the Chinese guidelines but inadequate TGWG by the IOM guidelines *vs.* women with optimal TGWG by both sets of the guidelines

Outcome	Optimal TGWG by both the Chinese and the IOM guidelines, n/N (%)	Optimal TGWG by the Chinese guidelines but inadequate TGWG by the IOM guidelines, n/N (%)	aOR (95% CI) ^b^
SGA	90/1094 (8.2)	47/428 (11.0)	1.36 (0.91, 2.02)
LGA	58/1094 (5.3)	12/428 (2.8)	0.51 (0.26, 0.99)
Respiratory distress	24/1094 (2.2)	19/428 (4.4)	1.07 (0.34, 3.32)
Neonatal jaundice	283/1094 (25.9)	130/428 (30.4)	1.09 (0.80, 1.49)
NICU admission	68/1094 (6.2)	43/428 (10.0)	1.00 (0.47, 2.11)
Any adverse outcome^a^	389/1094 (35.6)	180/428 (42.1)	1.06 (0.81, 1.40)

*Abbreviations: aOR* adjusted odds ratio, *ART* assisted reproductive technology, *CI* confidence interval, *GDM* gestational diabetes mellitus, *IOM* Institute of Medicine, *LGA* large for gestational age, *NICU* neonatal intensive care unit, *PBMI* prepregnancy body mass index, *SGA* small for gestational age, *TGWG*, total gestational weight gain

^a^Any adverse outcome was defined as the presence of one or more of the following neonatal outcomes: SGA, LGA, respiratory distress syndrome, neonatal jaundice, and NICU admission

^b^The models adjusted for the priori defined confounders, including maternal age, gestational age, maternal PBMI, parity, gravidity, education level, twin type, use of ART, historical cesarean section, family history of diabetes mellitus/hypertension, pre-existing diabetes mellitus/hypertension, GDM and gestational hypertension

## Discussion

In this retrospective cohort study of Chinese women with twin pregnancies, inadequate/excess TGWG identified by either the IOM or the Chinese guidelines, compared with the optimal TGWG, demonstrated statistically significantly higher risks of adverse neonatal outcomes. Inadequate TGWG according to either set of the guidelines was associated with increased risks of SGA birth and neonatal jaundice, while excess TGWG according to either set of the guidelines was associated with an increased risk of LGA birth. The agreement between the two sets of the guidelines was relatively high (Kappa coefficient = 0.721). Compared with women in the optimal TGWG group by both sets of the guidelines, women in the optimal by the Chinese guidelines but in the inadequate group by the IOM guidelines demonstrated no statistically significant difference in the risk for any adverse neonatal outcome studied.

The association between inappropriate TGWG and adverse neonatal outcomes among twin pregnancies in our study confirmed previous findings. The study of Guan, et al. showed that excess TGWG by IOM references was related to a higher risk of LGA, and inadequate TGWG was associated with increased risks of SGA [16]. A larger cohort study of twin pregnancies in China suggested that inadequate TGWG according to the IOM references was associated with higher risk of SGA in all PBMI groups [11].

The Chinese guidelines we investigated in the present study recommend lower TGWGs than do the IOM guidelines for North American women. Similarly, two studies among Japanese women also recommended lower TGWGs [9, 12]. This discrepancy may be explained by the ethnical difference in body size **─** Asian women are relatively short and slim compared with Caucasian women. Chinese women with optimal TGWG according to the Chinese guidelines could be classified as inadequate TGWG by the IOM guidelines because of this discrepancy, and these women accounted for 16.6% of our study population. Additionally, the IOM guidelines for twin pregnancies do not specify the optimal TGWG for underweight women due to lack of data [17]. In China, the prevalence of underweight before pregnancy was estimated to be 12%-17% [11, 18, 19]. In the present study, 16.0% of women were underweight before pregnancy, highlighting the need to determine the optimal TGWG for these women.

The relatively high agreement between the IOM and the Chinese guidelines and their similar associations with adverse neonatal outcomes suggest that the IOM guidelines are applicable to Chinese twin pregnancies. However, compared with those who were classified as optimal TGWG by both sets of the guidelines, women whose TGWG was classified as optimal by the Chinese guidelines but inadequate by the IOM guidelines did not have an increased risk of any adverse neonatal outcome, suggesting that the Chinese guidelines might be more suitable for Chinese women with twin pregnancies.

The present study was not designed to analyze the association between inappropriate TGWG and maternal prepartum complications such as GDM and preeclampsia. Although some of the previous studies have reported positive associations of inappropriate TGWG with such complications [5, 10], reliability of their results is questionable for the apparently indeterminable causal relationships.

This study is the first study that externally validated and compared the Chinese and the IOM guidelines in Chinese women with twin pregnancies. There are several limitations in this study. First of all, we recruited women with twin pregnancies who visited hospitals for prenatal examination other than those visited community health centers. Those who visit hospitals for prenatal examination may have higher socioeconomic status and might gain different weight from the others. Therefore, extrapolation of these results to all twin-pregnancy women should be performed with caution. Second, prepregnancy weight in this study was self-reported rather than measured, which may cause bias. However, we found a good correlation between self-reported prepregnancy weight and weight measured in the first 12 gestational weeks (correlation coefficient = 0.915, *n* = 324). Furthermore, this study focused on TGWG, but GWG rate (i.e., GWG per week), which takes into account different patterns of GWG throughout pregnancy might be more predictive of adverse neonatal outcomes. However, there has been no guidelines on GWG rate for twin pregnancies.

## Conclusions

Inappropriate TGWG defined by either the IOM or the Chinese guidelines was associated with increased risks of adverse neonatal outcomes, suggesting that both are applicable to Chinese women with twin pregnancies.

## Supplementary Information

**Additional file 1: Table S1.** Baseline characteristics of women with twin pregnancies.

**Additional file 2: Table S2.** Adjusted risk associations of neonatal outcomes for women with inadequate or excess TGWG vs. women with optimal TGWG, defined by the Chinese guidelines, after excluding underweight women.

## Data Availability

The datasets generated and/or analysed during the current study are not publicly available due to ethical restrictions, but are available from the corresponding author on reasonable request.

## Abbreviations

aOR: Adjusted Odds Ratios
ART: Assisted Reproductive Technology
GDM: Gestational Diabetes Mellitus
GEE: General Estimated Equations
GWG: Gestational Weight Gain
IOM: The Institute of Medicine
LGA: Large for Gestational Age
NICU: Neonatal Intensive Care Unit
SGA: Small for Gestational Age
TGWG: Total Gestational Weight Gain
