# Supplementary material for: Associations between gestational weight gain and adverse neonatal outcomes: a comparison between the US and the Chinese guidelines in Chinese women with twin pregnancies
Source: BMC Public Health. 2023 Jan 19;23:134. doi: 10.1186/s12889-023-15008-z (PMC9850551; doi:10.1186/s12889-023-15008-z)
Supplement: Supplementary file 1 — Additional file 1: Table S1. Baseline characteristics of women with twin pregnancies. [file 12889_2023_15008_MOESM1_ESM.docx]

**Table S1. Baseline characteristics of women with twin pregnancies.**

| **Characteristics** | The Chinese guidelines | | | The IOM guidelines | | | |
| --- | --- | --- | --- | --- | --- | --- | --- |
|  | Inadequate TGWG | Optimal TGWG | Excess TGWG | Mothers excluding underweight women | Inadequate TGWG | Optimal TGWG | Excess TGWG |
| N | 572 | 857 | 105 | 1289 | 651 | 558 | 80 |
| Maternal age, year, median [IQR] | 31.0[28.0-35.0] | 31.0[29.0-34.0] | 31.0[28.0-34.0] | 32.0[29.0-35.0] | 32.0[29.0-35.0] | 32.0[29.0-34.0] | 31.0[28.0-33.2] |
| Gestational age, weeks, median [IQR] | 36.0[34.0-37.0] | 37.0[36.0-37.0] | 37.0[36.0-37.0] | 37.0[35.0-37.0] | 36.0[34.0-37.0] | 37.0[36.0-37.0] | 37.0[36.0-37.0] |
| Nulliparity, n(%) | |  |  |  |  |  |  |
| No | 230(40.2) | 326(38.0) | 43(41.0) | 519(40.3) | 277(42.5) | 213(38.2) | 29(36.2) |
| Yes | 342(59.8) | 531(62.0) | 62(59.0) | 770(59.7) | 374(57.5) | 345(61.8) | 51(63.7) |
| Primigravida, n(%) | |  |  |  |  |  |  |
| No | 318(55.6) | 473(55.2) | 63(60.0) | 731(56.7) | 380(58.4) | 304(54.5) | 47(58.8) |
| Yes | 254(44.4) | 384(44.8) | 42(40.0) | 558(43.3) | 271(41.6) | 254(45.5) | 33(41.2) |
| Categorical PBMI, kg/m^2^, n(%)^a^ | |  |  |  |  |  |  |
| Underweight | 135(23.6) | 96(11.2) | 14(13.3) | - | - | - | - |
| Normal | 356(62.2) | 620(72.3) | 60(57.1) | 1108(86.0) | 562(86.3) | 481(86.2) | 65(81.2) |
| Overweight | 65(11.4) | 111(13.0) | 22(21.0) | 158(12.3) | 78(12.0) | 69(12.4) | 11(13.8) |
| Obese | 16(2.8) | 30(3.5) | 9(8.6) | 23(1.8) | 11(1.7) | 8(1.4) | 4(5.0) |
| Education level, n(%) | |  |  |  |  |  |  |
| Primary school or middle school | 8(1.4) | 9(1.1) | 1(1.0) | 16(1.2) | 8(1.2) | 7(1.3) | 1(1.2) |
| High school | 77(13.5) | 103(12.0) | 27(25.7) | 171(13.3) | 84(12.9) | 64(11.5) | 23(28.7) |
| College | 487(85.1) | 745(86.9) | 77(73.3) | 1102(85.5) | 559(85.9) | 487(87.3) | 56(70.0) |
| Use of ART, n(%) | |  |  |  |  |  |  |
| No | 259(45.3) | 386(45.0) | 58(55.2) | 583(45.2) | 302(46.4) | 239(42.8) | 42(52.5) |
| Yes | 313(54.7) | 471(55.0) | 47(44.8) | 706(54.8) | 349(53.6) | 319(57.2) | 38(47.5) |
| Historical cesarean section, n(%)^b^ | |  |  |  |  |  |  |
| No | 144(62.6) | 204(62.6) | 25(58.1) | 323(62.2) | 171(61.7) | 135(63.4) | 17(58.6) |
| Yes | 86(37.4) | 122(37.4) | 18(41.9) | 196(37.8) | 106(38.3) | 78(36.6) | 12(41.4) |
| Family history of diabetes mellitus/hypertension, n(%) | | | |  |  |  |  |
| No | 500(87.4) | 711(83.0) | 87(82.9) | 1086(84.3) | 551(84.6) | 466(83.5) | 69(86.2) |
| Yes | 72(12.6) | 146(17.0) | 18(17.1) | 203(15.7) | 100(15.4) | 92(16.5) | 11(13.8) |
| Pre-existing diabetes mellitus/hypertension, n(%) | | |  |  |  |  |  |
| No | 556(97.2) | 846(98.7) | 103(98.1) | 1261(97.8) | 631(96.9) | 550(98.6) | 80(100.0) |
| Yes | 16(2.8) | 11(1.3) | 2(1.9) | 28(2.2) | 20(3.1) | 8(1.4) | 0(0.0) |
| GDM, n(%) |  |  |  |  |  |  |  |
| No | 407(71.2) | 670(78.2) | 80(76.2) | 948(73.5) | 447(68.7) | 440(78.9) | 61(76.2) |
| Yes | 165(28.8) | 187(21.8) | 25(23.8) | 341(26.5) | 204(31.3) | 118(21.1) | 19(23.8) |
| Gestational hypertension, n(%) | |  |  |  |  |  |  |
| No | 494(86.4) | 711(83.0) | 76(72.4) | 1069(82.9) | 560(86.0) | 450(80.6) | 59(73.8) |
| Yes | 78(13.6) | 146(17.0) | 29(27.6) | 220(17.1) | 91(14.0) | 108(19.4) | 21(26.2) |
| Delivery mode, n(%) | |  |  |  |  |  |  |
| Cesarean section | 524(91.6) | 815(95.1) | 101(96.2) | 1211(93.9) | 605(92.9) | 529(94.8) | 77(96.2) |
| Vaginal delivery | 48(8.4) | 42(4.9) | 4(3.8) | 78(6.1) | 46(7.1) | 29(5.2) | 3(3.8) |
| Twin type, n(%) | |  |  |  |  |  |  |
| Dichorionic | 420(73.4) | 649(75.7) | 69(65.7) | 966(74.9) | 484(74.3) | 428(76.7) | 54(67.5) |
| Monochorionic-diamniotic | 149(26.0) | 205(23.9) | 35(33.3) | 317(24.6) | 163(25.0) | 128(22.9) | 26(32.5) |
| Monoamniotic | 3(0.5) | 3(0.4) | 1(1.0) | 6(0.5) | 4(0.6) | 2(0.4) | 0(0.0) |
| Offspring sex, n(%) | |  |  |  |  |  |  |
| Male | 602(52.6) | 903(52.7) | 104(49.5) | 1348(52.3) | 693(53.2) | 575(51.5) | 80(50.0) |
| Female | 542(47.4) | 811(47.3) | 106(50.5) | 1230(47.7) | 609(46.8) | 541(48.5) | 80(50.0) |

^a^ Categorical PBMI by Chinese criteria was used in the Chinese guidelines; Categorical PBMI by WHO criteria was used in the IOM guidelines.

^b^ The percentage of historical cesarean section was calculated in parous women.

Abbreviations: ART, assisted reproductive technology; GDM, gestational diabetes mellitus; IOM, Institute of Medicine; IQR, inter-quartile range; PBMI, prepregnancy body mass index; TGWG, total gestational weight gain; WHO, World Health Organization.
