# Supplementary material for: Associations between gestational weight gain and adverse neonatal outcomes: a comparison between the US and the Chinese guidelines in Chinese women with twin pregnancies
Source: BMC Public Health. 2023 Jan 19;23:134. doi: 10.1186/s12889-023-15008-z (PMC9850551; doi:10.1186/s12889-023-15008-z)
Supplement: Supplementary file 2 — Additional file 2: Table S2. Adjusted risk associations of neonatal outcomes for women with inadequate or excess TGWG vs. women with optimal TGWG, defined by the Chinese guidelines, after excluding underweight women. [file 12889_2023_15008_MOESM2_ESM.docx]

**Table S2. Adjusted risk associations of neonatal outcomes for women with inadequate or excess TGWG *vs.* women with optimal TGWG, defined by the Chinese guidelines, after excluding underweight women.**

|  | **Optimal TGWG ^b^** | **Inadequate TGWG** | | **Excess TGWG** | |
| --- | --- | --- | --- | --- | --- |
|  | **n/N (%)** | **n/N (%)** | **aOR (95% CI) ^c^** | **n/N (%)** | **aOR (95% CI) ^c^** |
| SGA | 137/1522 (9.0) | 118/974 (13.5) | 1.59 (1.17, 2.15) | 6/182 (3.3) | 0.33 (0.14, 0.77) |
| LGA | 70/1522 (4.6) | 23/974 (2.6) | 0.63 (0.37, 1.05) | 31/182 (17.0) | 4.44 (2.65, 7.46) |
| Respiratory distress | 43/1522 (2.8) | 87/974 (10.0) | 1.43 (0.80, 2.56) | 9/182 (4.9) | 1.78 (0.53, 5.97) |
| Neonatal jaundice | 413/1522 (27.1) | 329/974 (37.6) | 1.30 (1.03, 1.62) | 43/182 (23.6) | 0.77 (0.49, 1.21) |
| NICU admission | 111/1522 (7.3) | 124/974 (14.2) | 0.73 (0.46, 1.18) | 6/182 (3.3) | 0.21 (0.02, 1.89) |
| Any adverse outcome ^a^ | 569/1522 (37.4) | 447/974 (51.1) | 1.31 (1.07, 1.61) | 78/182 (42.9) | 1.20 (0.83, 1.75) |

^a^ Any adverse outcome was defined as the presence of one or more of the following neonatal outcomes: SGA, LGA, respiratory distress syndrome, neonatal jaundice, and NICU admission.

^b^ Optimal TGWG was used as the reference.

^c^ All the models adjusted for the priori defined confounders, including maternal age, gestational age, maternal PBMI, parity, gravidity, education level, twin type, use of ART, historical cesarean section, family history of diabetes mellitus/hypertension, pre-existing diabetes mellitus/hypertension, GDM and gestational hypertension.

Abbreviations: aOR, adjusted odds ratio; ART, assisted reproductive technology; CI, confidence interval; GDM, gestational diabetes mellitus; LGA, large for gestational age; NICU, neonatal intensive care unit; PBMI, prepregnancy body mass index; SGA, small for gestational age; TGWG, total gestational weight gain.
